# Combination of PD-1/PD-L1 and CTLA-4 inhibitors in the treatment of cancer – a brief update

**DOI:** 10.3389/fimmu.2025.1680838

**Published:** 2025-10-13

**Authors:** Johan Park, Bjørn Steen Skålhegg

**Affiliations:** Division for Molecular Nutrition, Institute for Basic Medical Sciences, University of Oslo, Oslo, Norway

**Keywords:** immunotherapy, immune checkpoint inhibitor, PD- 1/L1, CTLA- 4, cancer

## Abstract

The introduction of immune checkpoint inhibitors (ICIs) has revolutionized cancer therapy, offering durable responses in multiple malignancies by targeting regulatory pathways such as programmed cell death protein 1 (PD-1), its ligand (PD-L1), and cytotoxic T-lymphocyte-associated protein 4 (CTLA-4). These pathways, which normally maintain immune tolerance and homeostasis, can be exploited by tumors to evade immune surveillance. Dual blockade using monoclonal antibodies (mAbs) targeting PD-1/PD-L1 and CTLA-4 has shown synergistic effects, improving response rates, overall survival, and progression-free survival in several cancer types. The U.S. Food and Drug Administration (FDA) has approved two such combinations: nivolumab plus ipilimumab and durvalumab plus tremelimumab, based on demonstrated clinical benefit in melanoma, renal cell carcinoma, colorectal cancer, hepatocellular carcinoma, non-small cell lung cancer, pleural mesothelioma, and esophageal squamous cell carcinoma. However, clinical benefit has not been consistent across all tumor types, with limited efficacy observed in cancers such as glioblastoma, head and neck squamous cell carcinoma, and Merkel cell carcinoma. Additionally, combination therapy is associated with a higher incidence of immune-related adverse events, affecting multiple organ systems and necessitating careful dosing strategies to balance efficacy and toxicity. This review summarizes the current landscape of FDA-approved PD-1/PD-L1 and CTLA-4 combinations, their therapeutic achievements, clinical limitations, and supports future research in combination immunotherapy.

## Background

With the understanding of cancer as a genomic disease, it is recognized that oncogenesis can lead to the formation of tumor-specific neoantigens (1). These molecular markers distinguish malignant cells from normal tissue. These neoantigens are captured by dendritic cells (DC) and presented to T cells, potentially triggering a cascade of immune responses, perceiving the cancer cells as foreign entities (1–3). However, cancer cells can develop mechanisms to evade this immune surveillance by exploiting regulatory pathways such as immune checkpoints. These checkpoints normally maintain immune tolerance and prevent autoimmunity, but in the context of cancer, they contribute to immune escape (1, 4). This dynamic interaction between the immune system and tumor progression is encapsulated by the theory of cancer immunoediting (1, 5). Immune checkpoints include co-stimulatory (e.g., CD27, CD28, CD40, CD137, ICOS, GITR, 4-1BB, OX-40) and co-inhibitory (e.g., PD-1, CTLA-4) molecules, which respectively enhance or suppress T-cell activation (6–8). Cancer cells can exploit the inhibitory pathway within the tumor microenvironment (TME), to promote immunosuppression and support disease progression (9, 10). This insight led to the development of immune checkpoint inhibitors (ICIs) targeting programmed cell death protein 1 (PD-1) and cytotoxic T-lymphocyte-associated protein 4 (CTLA-4), which aim to restore T-cell activity and reinvigorate antitumor immunity (11).

The advent of ICIs has marked a transformative shift in cancer therapy, offering durable responses across a wide range of malignancies. Among the most extensively studied and clinically impactful targets are PD-1, its ligand PD-L1, and CTLA-4. To date, thirteen FDA-approved agents targeting PD-1, PD-L1 or CTLA-4 are used in the treatment of multiple cancer types, including melanoma, lung cancer, lymphoma, gastric cancer, Merkel cell carcinoma, head and neck cancer, hepatocellular carcinoma, cervical cancer, urothelial cancer, renal cell cancer, biliary tract cancer, cutaneous squamous cell cancer, endometrial cancer, breast cancer, and sarcoma (**Table 1**).

**Table 1:** List of FDA approved drugs targeting PD-1, PD-L1, CTLA-4 and their combinations (current as July 2025).

Drug	Brand name	Year of first approval	Indication
PD-1 inhibitors			
- Pembrolizumab - Nivolumab - Cemiplimab - Dostarlimab - Retifanlimab - Toripalimab - Tislelizumab	Keytruda Opdivo Libtayo Jemperli Zynyz Loqtorzi Tevimbra	2014 2014 2018 2021 2023 2023 2024	Metastatic melanoma, NSCLC, HNSCC, classical Hodgkin's lymphoma, primary mediastinal B-cell lymphoma, UC, gastric cancer, cervical cancer, HCC, Merkel cell carcinoma, RCC, SCLC, ESCC, endometrial cancer, cutaneous squamous cell carcinoma, biliary tract cancer (w/ gemcitabine + cisplatin), malignant pleural mesothelioma (w/ pemetrexed + platinum) Metastatic melanoma, NSCLC, RCC, classical Hodgkin's lymphoma, HNSCC, UC, colorectal cancer, HCC, SCLC, ESCC, gastric cancer (or gastroesophageal junction cancer), Cutaneous squamous cell carcinoma, basal cell carcinoma, NSCLC Endometrial cancer, solid tumor Merkel cell carcinoma, anal squamous cell carcinoma (w/ carboplatin + paclitaxel) Nasopharyngeal carcinoma ESCC
PD-L1 inhibitors			
- Atezolizumab - Avelumab - Durvalumab - Cosibelimab	Tecentriq Bavencio Imfinzi Unloxcyt	2016 2017 2017 2024	UC, NSCLC, SCLC (w/ carboplatin + etoposide), HCC (w/ bevacizumab), metastatic melanoma (w/ cobimetinib + vemurafenib), sarcoma Merkel cell carcinoma, UC, RCC UC, NSCLC, SCLC, biliary tract cancer (w/ gemcitabine + cisplatin), endometrial cancer (w/ carboplatin + paclitaxel), bladder cancer (w/ gemcitabine + cisplatin) Cutaneous squamous cell carcinoma
CTLA-4 inhibitors			
- Ipilimumab - Tremelimumab	Yervoy Imjuno	2011 2022	Metastatic melanoma HCC (w/ durvalumab), NSCLC (w/ durvalumab + chemotherapy)
Combination of CTLA-4 and PD-1 inhibitor			
- Ipilimumab + nivolumab	Yervoy + Opdivo	2015	Metastatic melanoma, RCC, colorectal cancer, HCC, NSCLC, malignant pleural mesothelioma, ESCC
Combination of CTLA-4 and PD-L1 inhibitor			
- Tremelimumab + Durvalumab	Imjuno + Imfinzi	2022	HCC

Data adapted from FDA (20, 21).

While monotherapy with PD-1, PD-L1 or CTLA-4 inhibitors has demonstrated clinical benefit, response rates remain limited, and only a subset of patients experience durable outcomes (12). Combining inhibitors of PD-1/PD-L1 and CTLA-4 was therefore of considerable interest, offering a mechanistic rationale for enhancing antitumor immunity via complementary actions: amplifying T-cell priming in lymphoid tissues and reversing immunosuppression within the TME (13–17). The mechanistic basis for these targets is discussed in the following sections. Dual immune checkpoint blockade has shown synergistic activity in preclinical models (12, 18, 19) and improved outcomes in several clinical settings. This review presents and discusses results from clinical studies evaluating PD-1/PD-L1 and CTLA-4 inhibitor combinations, highlighting current achievements, clinical limitations, and future directions in cancer immunotherapy. Notably, this review focuses exclusively on ICI combinations targeting PD-1/PD-L1 and CTLA-4 that have received FDA approval; investigational agents or drugs approved by others are beyond its scope.

### CTLA-4

CTLA-4 — also known as CD152 — is a co-inhibitory receptor primarily expressed on activated T cells (CD4+ and CD8+) and regulatory T cells (Tregs), and it functions during the early stages of T-cell activation in secondary lymphoid organs (22–24). Upon antigen presentation, naïve T cells require two signals for full activation: (1) T-cell antigen receptor (TCR) recognition of antigen-MHC complexes, and (2) co-stimulatory signaling via CD28 binding to B7 ligands such as B7-1/CD80 dimer and B7-2/CD86 monomer on antigen-presenting cells (APCs) (25, 26). CTLA-4 competes with CD28 for binding to these B7 ligands but with significantly higher affinity, thereby outcompeting CD28 and delivering an inhibitory signal that attenuates T-cell activation and proliferation (27–29). Additionally, CTLA-4 enhances the suppressive activity of Tregs and reduces the availability of B7 ligands on APCs through trans-endocytosis (30, 31). This contributes to an immunosuppressive environment, particularly in lymph nodes where T-cell priming occurs.

CTLA-4 blockade, using monoclonal antibodies (mAbs), disrupts this inhibitory interaction, thereby augmenting T-cell activation, promoting clonal expansion, and enhancing effector and memory T-cell development (32). To date, ipilimumab has been approved by the FDA as monotherapy for the treatment of melanoma (**Table 1**).

### PD-1/PD-L1

PD-1 — also known as CD279 — is another co-inhibitory receptor primarily expressed on activated T cells, but also present on B cells, natural killer (NK) cells, myeloid cells, monocytes, neutrophils, and DCs (33, 34). Similar to CTLA-4, PD-1 inhibits T-cell activation; however, it predominantly functions during later stages of the immune response. It plays a key role in suppressing differentiated effector T cells in peripheral tissues and within the tumor microenvironment (TME). The TME is considered the solid tumor body and for cancers it contains a vast number of molecules and cells base on composition that will characterize growth expansion and metastatic potential of a solid tumor (16, 34–37). PD-1 has two ligands: PD-L1 (B7-H1/CD274) and PD-L2 (B7-DC/CD273). PD-L1 is widely expressed on both hematopoietic cells, including T cells, B cells, DCs, and macrophages (Mø), and non-hematopoietic cells such as vascular endothelial cells, stromal cells, pancreatic islet cells, placental syncytiotrophoblasts, and keratinocytes. PD-L2 expression is more restricted and primarily found on APCs (33, 38, 39). Both ligands can be expressed by cancer cells, although PD-L1 is more commonly upregulated. Its expression can also be induced by inflammatory cytokines such as type I and type II IFNs, TNF-α, and IL-6, enabling tumors to escape immune destruction through adaptive immune resistance (35, 38, 40, 41). Engagement of PD-1 by its ligands recruits SHP-1 and SHP-2 tyrosine phosphatases, which dephosphorylate downstream signaling molecules involved in the TCR and CD28 pathways (42, 43). This leads to reduced cytokine production, proliferation, and cytotoxic function of T cells, and promotes T-cell exhaustion during chronic antigen exposure (44).

Cancer cells exploit this mechanism by overexpressing PD-L1 on their surface. At the same time, PD-1 is highly expressed on tumor-infiltrating T cells, including exhausted T cells within the TME. This interaction allows cancer cells to evade immune surveillance and suppress effective antitumor immune responses. mAbs targeting PD-1 or PD-L1 have been developed to block this interaction, thereby restoring effector T-cell function and promoting antitumor immunity. To date, seven PD-1 inhibitors and four PD-L1 inhibitors have received FDA approval for the treatment of various cancers (**Table 1**).

## Clinical data

### Melanoma

#### Nivolumab + ipilimumab in melanoma

Combined checkpoint inhibition with nivolumab (PD-1 inhibitor) and ipilimumab (CTLA-4 inhibitor) in metastatic melanoma has been extensively studied in multiple clinical trials (45–47, 56–71). Given the enhanced antitumor activity observed in preclinical models compared to monotherapy (18), nivolumab combined with ipilimumab was first tested in advanced melanoma through a phase 1 trial. The combination produced high response rates of up to 40%, with rapid and deep responses; 31% of patients experienced ≥80% tumor reduction within 12 weeks (45). A subsequent phase 2 study compared the combination to ipilimumab alone in treatment-naive patients with unresectable or metastatic melanoma. In BRAF V600 wild-type patients, the combination achieved an overall response rate (ORR) of 61.1% versus 10.8%; in those with BRAF mutations, ORR was 52.2% versus 10.0%. Median progression-free survival (PFS) was not reached in the wild-type group and was 8.5 months in the BRAF-mutant group. The hazard ratios (HR) for disease progression were 0.40 (p<0.001) and 0.38, respectively (46). These findings led to the FDA’s accelerated approval of the combination in 2015 for patients with unresectable or metastatic BRAF V600 wild-type melanoma (**Table 2**). A follow-up analysis reported a 2-year overall survival (OS) rate of 63.8%, with median OS not yet reached at the time of analysis (56). The CheckMate 067 phase 3 trial later compared the combination to nivolumab and ipilimumab monotherapies in patients with unresectable or metastatic melanoma. Initial results showed a significantly improved median PFS of 11.5 months and an ORR of 57.6% with the combination, versus 6.5 months and 43.7% with nivolumab, and 2.9 months and 19% with ipilimumab (47). Long-term follow-ups confirmed sustained benefit, with OS rates of 58%, 52%, and 43% at 3, 5, and 10 years, respectively, and a median OS of 71.9 months (57–61). Despite the durable efficacy, treatment with the combination was associated with a high rate of toxicity, with grade 3–4 adverse events occurring in 59% of patients (61). To address this, a phase 3b/4 trial evaluated alternative dosing strategies aimed at improving tolerability. The regimen of nivolumab 3 mg/kg plus ipilimumab 1 mg/kg (NIVO3+IPI1) resulted in a significantly lower rate of grade 3–4 AEs compared to the standard nivolumab 1 mg/kg plus ipilimumab 3 mg/kg (NIVO1+IPI3) regimen (34% vs 48%), while maintaining comparable efficacy. ORR was 45.6% versus 50.6%, complete response rates were 15.0% and 13.5%, and median PFS was 9.9 versus 8.9 months, respectively (62). A subsequent study confirmed these findings, supporting NIVO3+IPI1 as a less toxic yet effective alternative (63). However, further investigation is warranted to confirm long-term clinical equivalence. Of note, multiple studies have shown that combination of ipilimumab and nivolumab appears significantly less effective than ipilimumab monotherapy in patients that progressed on PD-1 inhibition therapy in melanoma (64, 65).

**Table 2:** Clinical trials supporting FDA approvals of the combination.

Patient group	Treatment	Year of approval	Trial, ID
BRAF V600 WILD-TYPE, UNRESECTABLE OR METASTATIC MELANOMA	Nivolumab + ipilimumab	2015	Phase 2, NCT01927419 (CheckMate 069)
ADVANCED RENAL CELL CARCINOMA	Nivolumab + ipilimumab	2018	Phase 3, NCT02231749 (CheckMate 214)
COLORECTAL CANCER WITH MSI-H/dMMR	Nivolumab + ipilimumab	2018	Phase 2, NCT02060188 (CheckMate 142)
HEPATOCELLULAR CARCINOMA	Nivolumab + ipilimumab	2020	Phase 1/2, NCT01658878 (CheckMate 040)
METASTATIC NON-SMALL CELL LUNG CARCINOMA (PD-L1 TUMOR EXPRESSION ≥1%)	Nivolumab + ipilimumab	2020	Phase 3, NCT02477826 (CheckMate 227)
UNRESECTABLE MALIGNANT PLEURAL MESOTHELIOMA	Nivolumab + ipilimumab	2020	Phase 3, NCT02899299 (CheckMate 743)
ESOPHAGEAL SQUAMOUS CELL CARCINOMA	Nivolumab + ipilimumab	2022	Phase 3, NCT03143153 (CheckMate 648)
UNRESECTABLE HEPATOCELLULAR CARCINOMA	Durvalumab + tremelimumab	2022	Phase 3, NCT03298451 (HIMALAYA)
HEPATOCELLULAR CARCINOMA	Nivolumab + ipilimumab	2025	Phase 3, NCT04039607 (CheckMate 9DW)
COLORECTAL CANCER WITH MSI-H/dMMR	Nivolumab + ipilimumab	2025	Phase 3, NCT04008030 (CheckMate 8HW)

The efficacy of nivolumab plus ipilimumab has also been evaluated in patients with melanoma brain metastases (MBM) (**Table 3**) (48, 49, 72–76). A multicenter phase 2 trial assessed the combination in both asymptomatic and symptomatic patients. In the asymptomatic cohort, outcomes were favorable, with an ORR of 53.5%, intracranial clinical benefit of 57.4%, 36-month intracranial PFS of 54.1%, and 36-month OS of 71.9%. In contrast, efficacy was limited in symptomatic patients, with respective rates of 16.7%, 16.7%, 18.9%, and 36.6% (48). Another multicenter phase 2 study in asymptomatic MBM compared nivolumab monotherapy to the combination, reporting higher intracranial response with the combination (46% vs 20%) (72). At 7-year follow-up, this benefit persisted, with intracranial PFS of 42% vs 15% and OS of 48% vs 26% (73). In a phase 3 trial, the combination was compared with fotemustine, demonstrating superior survival: median OS of 29.2 vs 8.5 months, 4-year OS of 41.0% vs 10.9%, and a HR for death of 0.44 (95% CI, 0.22–0.87; p = 0.017) (49). The 7-year OS remained durable at 42.8% vs 10.0% (74).

**Table 3:** Clinical studies in melanoma.

*Patient group*	*Treatment*	*Participants (n)*	*Trial, ID*	*Outcomes*	*Reference*
*Advanced melanoma*	Nivolumab + ipilimumab	53	Phase 1, NCT01024231	**ORR:** 40% **≥80% tumor reduction at 12 weeks:** 31% **Grade 3–4 AE:** 53%	Wolchok et al., 2013 (45)
*Advanced melanoma*	Nivolumab + ipilimumab	95 (BRAF wild-type=72, BRAF V600 mutant=23)	Phase 2, NCT01927419 (CheckMate 069)	**ORR:** 61.1% | 52.2% **ORR in PD-L1-positive:** 58% **ORR in PD-L1 negative:** 55% **pCR:** 22.2% | 21.7% **Median PFS:** Not reached | 8.5 months **Any grade AEs:** 91% **Grade 3–4 AEs:** 54%	Postow et al., 2015 (46)
*Previously untreated metastatic melanoma*	Nivolumab + ipilimumab	314	Phase 3, NCT01844505 (CheckMate 067)	**ORR:** 57.6% **Median PFS:**11.5 months **Median PFS in wild-type BRAF:** 11.2 months **Median PFS in BRAF mutation:** 11.7 months **Median PFS in PD-L1 positive:** 14.0 months **Median PFS in PD-L1 negative:** 11.2 months **12 months PFS rate:** 53% **Grade 3–4 AEs:** 55.0%	Larkin et al., 2015 (47)
*Melanoma brain metastases*	Nivolumab + ipilimumab	119 (asymptomatic=101, symptomatic=18)	Phase 2, NCT02320058 (CheckMate 204)	**ORR:** 53.5% | 16.7% **Intracranial clinical benefit:** 57.4% | 16.7% **Median intracranial PFS by investigator assessment:** Not reached | 1.2 months **36-month intracranial PFS:** 54.1% | 18.9% **36-month OS:** 71.9% | 36.6% **Grade 3–4 AE:** 55% | 67%	Tawbi et al., 2021 (48)
*Asymptomatic melanoma brain metastases*	Nivolumab + ipilimumab	27	Phase 3, NCT02460068 (NIBIT-M2)	**Median OS: 29.2 months** **4-year OS: 41.0%**	Di Giacomo et al., 2021 (49)
*Resected stage IV melanoma*	Nivolumab + ipilimumab	56	Phase 2, NCT02523313	**Median RFS:** Not reached **1-year RFS:** 75% **2-year RFS:** 70% **Grade 3–4 AE:** 71%	Zimmer et al., 2020 (50)
*Resected stage IIIB-D/stage IV melanoma*	Nivolumab + ipilimumab	920	Phase 3, NCT03068455 (CheckMate 915)	**Median RFS:** Not reached **2-year RFS:** 64.6% **Median OS:** Not reached **2-year OS:** 89.9% **Median DMFS:** Not reached **2-year DMFS:** 75.4% **Grade 3–4 AE:** 32.6%	Weber et al., 2022 (51)
*Resectable stage III melanoma*	Nivolumab + ipilimumab	212	Phase 3, NCT04949113	**Estimated 12-month event free survival:** 83.7% **MPR:** 59% **Grade 3–4 AE:** 29.7%	Blank et al., 2024 (52)
*Metastatic uveal melanoma*	Nivolumab + ipilimumab	33	Phase 2, NCT01585194 (PROSPER)	**ORR:** 18% **Median PFS:** 5.5 months **Median OS:** 19.1 months **1-year OS:** 56% **Grade 3–4 AE:** 40%	Pelster et al., 2020 (53)
*Metastatic uveal melanoma*	Nivolumab + ipilimumab	52	Phase 2, NCT02626962 (GEM-1402)	**ORR:** 11.5% **Median PFS:** 3.0 months **6-month PFS:** 28.8% **12-month PFS:** 19.2% **Median OS:** 12.7 months **12-month OS:** 51.9% **24-month OS:** 26.4% **Grade ≥ 3 AE:** 57.7%	Piulats et al., 2021 (54)
*Advanced melanoma*	Pembrolizumab + ipilimumab	153	Phase 1b, NCT02089685 (KEYNOTE-029)	**ORR:** 61% **Median PFS:** Not reached **1-year PFS:** 69% **Median OS:** Not reached **1-year OS:** 89% **Grade 3–4 AE:** 45%	Long et al., 2017 (55)

AE, adverse event; DMFS, distant metastasis-free survival; MPR = major pathological response; ORR, objective response rate; OS, overall survival; pCR, pathological complete response; PFS, progression-free survival; RFS, recurrence-free survival.

The combination has also been investigated in the adjuvant setting (**Table 3**). A randomized, double-blind phase 2 trial evaluated nivolumab plus ipilimumab, nivolumab monotherapy, and placebo in patients with resected stage IV melanoma and no evidence of disease. Both active treatments significantly improved recurrence-free survival (RFS) compared to placebo, with 2-year RFS rates of 70% for the combination, 42% for monotherapy, and 14% for placebo (50). However, a subsequent phase 3 trial showed no additional benefit of the combination over nivolumab alone in RFS (64.5% vs 63.2%), OS (2-year: 89.8% vs 91.8%), or DMFS (2-year: 75.4% vs 77.4%) (51).

In the neoadjuvant setting, a phase 1b trial in high-risk stage III melanoma compared NIVO1+IPI3 given entirely postoperatively versus split pre-/postoperative administration. Neoadjuvant treatment achieved pathological responses in 78% (7/9) and greater expansion of tumor-resident T-cell clones, but 9/10 patients experienced severe AEs (77). A phase 2 trial comparing neoadjuvant nivolumab monotherapy and NIVO1+IPI3 in stage III or oligometastatic stage IV melanoma showed higher ORR with the combination (73% vs 25%), but increased grade 3–4 toxicity (73% vs 8%) (78). To optimize dosing, another phase 2 study tested three neoadjuvant regimens in macroscopic stage III melanoma: NIVO1+IPI3 (Arm A), NIVO3+IPI1 (Arm B), and IPI3 alone (Arm C). Arm B had the lowest grade 3–4 AE rate (20% vs 40% in A and 50% in C) and comparable radiologic (57% vs 63%) and pathologic response rates (77% vs 80%) to Arm A; Arm C performed worst (79). A subsequent phase 3 trial compared two cycles of neoadjuvant NIVO3+IPI1 to twelve cycles of adjuvant nivolumab monotherapy in resectable stage III melanoma. Patients in the neoadjuvant arm with incomplete response received additional adjuvant therapy. The 12-month event-free survival was 83.7%, with a HR for progression, recurrence, or death of 0.32 (99.9% CI, 0.15–0.66), supporting neoadjuvant NIVO3+IPI1 and response-driven adjuvant therapy as a promising strategy (52).

The nivolumab–ipilimumab combination has also been evaluated in metastatic uveal melanoma in two small phase 2 studies (**Table 3**). Despite limited sample sizes, both trials reported promising outcomes: ORR of 18% and 11.5%, median PFS of 5.5 and 3.0 months, median OS of 19.1 and 12.7 months, and 1-year OS rates of 56% and 51.9%. Both studies support the combination as an active option for this difficult-to-treat population, warranting further investigation in larger trials (53, 54).

#### Pembrolizumab + ipilimumab in melanoma

The combination of pembrolizumab and ipilimumab has been evaluated in metastatic or unresectable stage III/IV melanoma (**Table 3**). In a phase 1b trial, patients received pembrolizumab (2 mg/kg) with ipilimumab (1 mg/kg) every 3 weeks for four doses, followed by pembrolizumab monotherapy. The regimen showed promising efficacy, with an ORR of 61%, 1-year PFS of 69%, and 1-year OS of 89%, while grade 3–4 adverse events occurred in 45% of patients, with no treatment-related deaths (55). Separately, a phase 2 study evaluated the combination in advanced melanoma after progression on prior anti-PD-1/L1 therapy. The results showed promising antitumor activity and manageable toxicity. Median PFS was 5.0 months, and median OS was 24.7 months. Grade 3–4 adverse events occurred in 27% of patients (80).

### Renal cell carcinoma

Treatment with nivolumab plus ipilimumab in metastatic renal cell carcinoma was first explored in a phase 1 study testing two dosing regimens: NIVO3+IPI1 and NIVO1+IPI3. Both demonstrated comparable ORR (40.4%) and 2-year OS (67.3% vs 69.6%), but higher-grade adverse events were more frequent in the NIVO1+IPI3 arm (61.7% vs 38.3%) (81). Based on these results, the NIVO3+IPI1 regimen was selected for the phase 3 CheckMate 214 trial. The combination yielded an ORR of 42% (with 9% complete responses), median PFS of 11.6 months, and 18-month OS of 75%. Compared to sunitinib, the incidence of disease progression or death was significantly reduced (HR 0.63, p<0.001), although the improvement in PFS did not meet the prespecified threshold for statistical significance (HR 0.82, p=0.03) (82). These results led to FDA approval of nivolumab plus ipilimumab for advanced RCC in 2018 (**Table 2**). A subsequent analysis of patient-reported outcomes showed fewer symptoms and improved quality of life in the combination arm, and extended 4- and 8-year follow-ups confirmed its sustained efficacy and safety advantage over sunitinib (87–89). Additional studies have since reinforced the clinical benefit of this combination in advanced RCC (83). Separately, a study investigated an alternative strategy of nivolumab induction followed by nivolumab plus ipilimumab boosts, but overall efficacy appeared inferior to the upfront combination approach (90).

The combination has been evaluated on advanced non-clear-cell renal cell carcinoma (nccRCC), a group underrepresented in major RCC trials, in two studies (**Table 4**). In CheckMate 920, the combination showed an ORR of 19.6%, median PFS of 3.7 months, and median OS of 21.2 months, with 23% grade 3–4 adverse events (84). A smaller retrospective study (n=18) reported an ORR of 33.3% and median PFS of 7.1 months, with 38% grade 3–4 toxicity (85). The authors supported its use as a first-line option in advanced nccRCC (84, 85). The sequential use of ipilimumab added to nivolumab monotherapy was not supported in a phase 2 trial (86).

**Table 4:** Clinical studies in renal cell carcinoma.

*Patient group*	*Treatment*	*Participants (n)*	*Trial, ID*	*Outcomes*	*Reference*
*Metastatic RCC*	Nivolumab + ipilimumab	94 (NIVO3+IPI1^1^=47, NIVO1+IPI3^2^=47)	Phase 1, (CheckMate 016)	**ORR:** 40.4% | 40.4% **2-year OS**: 67.3% | 69.6% **Grade 3–4 AE:** 38.3% | 61.7%	Hammers et al., 2017 (81)
*Advanced RCC*	Nivolumab + ipilimumab	550	Phase 3, NCT02231749 (CheckMate 214)	**ORR:** 42% **12-month OS rate:** 80% **18-month OS rate:** 75% **Median OS:** Not reached **Median PFS:** 11.6 months **Any grade AEs:** 93% **Grade 3–4 AEs:** 46%	Motzer et al., 2018 (82)
*Advanced RCC*	Nivolumab + ipilimumab	30	Phase 2, NCT02996110 (FRACTION-RCC)	**ORR:** 20% **Median OS:** 38.2 months **1-year OS rate:** 84% **Median PFS:** 3.4 months **24-week PFS rate:** 49%	Choueiri et al., 2024 (83)
*Advanced non-clear-cell RCC*	Nivolumab + ipilimumab	52	Phase 3b/4, NCT02982954 (CheckMate 920)	**ORR:** 19.6% **Median PFS:** 3.7 months **Median OS:** 21.2 months **Grade 3–4 AE:** 23%	Tykodi et al., 2022 (84)
*Metastatic non-clear-cell RCC*	Nivolumab + ipilimumab	18	—	**ORR:** 33.3% **Median PFS:** 7.1 months **6-months PFS:** 60.6% **12-months PFS:** 46.8% **12-months OS:** 64.2% **Grade 3–4 AE:** 38%	Gupta et al., 2020 (85)
*Advanced non-clear- cell RCC*	Nivolumab + ipilimumab	41	Phase 2, NCT03177239 (UNISoN)	**ORR:** 10% **Median DOR:** 13.5 months **Median PFS:** 2.6 months **Grade ≥3 AE:** 49%	Conduit et al., 2024 (86)

^1^Nivolumab 3 mg/kg plus ipilimumab 1 mg/kg.

^2^Nivolumab 1 mg/kg plus ipilimumab 3 mg/kg. AE, adverse event; DOR, duration of response; ORR, objective response rate; OS, overall survival; PFS, progression-free survival.

### Colorectal cancer

Concurrent administration of nivolumab and ipilimumab was first evaluated as second-line therapy for DNA mismatch repair–deficient (dMMR) and microsatellite instability–high (MSI-H) metastatic colorectal cancer in a multicenter, open-label phase 2 trial (**Table 5**). This followed earlier findings in which nivolumab monotherapy showed clinical benefit (ORR 31%, disease control rate 69%, 12-month OS 73%) (95). In the combination cohort, patients received nivolumab 3 mg/kg plus low-dose ipilimumab 1 mg/kg. The regimen yielded an investigator-assessed ORR of 55% and a disease control rate of 80%. PFS rates at 9 and 12 months was 76% and 71%, respectively, while OS rates were 87% and 85%. Grade 3–4 treatment-related adverse events were reported in 32% of patients (91). Based on these results, the combination received accelerated FDA approval in 2018 for previously treated dMMR/MSI-H metastatic colorectal cancer (96), establishing dual checkpoint blockade as a promising strategy for this biomarker-defined population (**Table 2**).

**Table 5:** Clinical studies in colorectal cancer.

*Patient group*	*Treatment*	*Participants (n)*	*Trial, ID*	*Outcomes*	*Reference*
*Colorectal cancer with MSI-H and dMMR*	Nivolumab + ipilimumab	82	Phase 2, NCT02060188 (CheckMate 142)	**Investigator-assessed ORR:** 55% **9-month PFS rate:** 76% **12-month PFS rate:** 71% **9-month OS rate:** 87% **12-month OS rate:** 85% **Any grade AEs:** 73% **Grade 3–4 AEs:** 32%	Overman et al., 2018 (91)
*Colorectal cancer with MSI-H and dMMR*	Nivolumab + ipilimumab	202	Phase 3, NCT04008030 (CheckMate 8HW)	**Mean survival time at 24 months:** 19.2 months **12-month PFS rate:** 79% **24-month PFS rate:** 72% **Grade 3–4 AE:** 48%	André et al., 2024 (92)
*Colorectal cancer with MSI-H and dMMR*	Nivolumab + ipilimumab	354	Phase 3, NCT04008030 (CheckMate 8HW)	**ORR:** 71% **Median PFS:** Not reached **12-month PFS:** 76% **24-month PFS:** 71% **36-month PFS:** 68% **Grade 3–4 AE:** 22%	André et al., 2025 (93)
*dMMR and pMMR colon cancer*	Nivolumab + ipilimumab	35 (dMMR=20, pMMR=15)	Phase 2, NCT03026140 (NICHE)	**Pathological response:** 100% | 27% **MPR:** 95% | 20% **pCR:** 60% | 13%	Chalabi et al., 2020 (94)

AE, adverse event; dMMR, mismatch repair deficient; MPR, major pathological response; ORR, objective response rate; OS, overall survival; pCR, pathological complete response; PFS, progression-free survival; pMMR, mismatch repair–proficient.

First-line treatment with nivolumab and ipilimumab for patients with dMMR and MSI-H metastatic colorectal cancer was evaluated in two phase 3 trials comparing the combination to either chemotherapy or nivolumab monotherapy (**Table 5**). In the first study, nivolumab plus ipilimumab significantly outperformed chemotherapy in progression-free survival, with 12- and 24-month PFS rates of 79% and 72%, respectively, versus 21% and 14% with chemotherapy. The restricted mean survival time at 24 months was 19.2 months (95% CI, 17.9–20.5) with the combination, compared to 8.6 months (95% CI, 6.7–10.4) with chemotherapy, a difference of 10.6 months (95% CI, 8.4–12.9) (92). The second study demonstrated superior efficacy for nivolumab plus ipilimumab compared to nivolumab monotherapy, with an ORR of 71% versus 58% and median PFS not reached versus 39.3 months. PFS rates at 12, 24, and 36 months were 76%, 71%, and 68% with the combination, versus 63%, 56%, and 51% with nivolumab alone. The HR for progression was 0.62 (95% CI, 0.48–0.81; p = 0.0003) (93). These findings supported FDA approval of the combination in 2025 as a first-line treatment for dMMR/MSI-H metastatic colorectal cancer (**Table 2**) (97).

The feasibility and safety of combining nivolumab and ipilimumab in the neoadjuvant setting for early-stage colon cancer were evaluated in a phase 2 study, including both mismatch repair–proficient (pMMR) and mismatch repair–deficient (dMMR) tumors. Pathologic responses were observed in all dMMR tumors, with 95% (19/20) achieving major pathologic responses and 60% (12/20) achieving complete pathologic responses. In contrast, 24% of pMMR tumors showed pathologic response, including 20% with major responses and 13% with complete responses. The study also found that infiltration by CD8^+^PD-1^+^ T cells was predictive of response in pMMR tumors. Authors concluded that the combination may represent a potential standard of care for selected patients with colon cancer and warrants further investigation in larger trials (94).

### Hepatocellular carcinoma

#### Nivolumab + ipilimumab in HCC

The combination of nivolumab and ipilimumab was initially investigated as a second-line treatment for unresectable hepatocellular carcinoma (HCC) in a phase 1/2 study. The trial evaluated three dosing regimens in patients previously treated with sorafenib. Arm A received nivolumab 1 mg/kg plus ipilimumab 3 mg/kg every 3 weeks for four doses; Arm B received nivolumab 3 mg/kg plus ipilimumab 1 mg/kg on the same schedule; and Arm C received nivolumab 3 mg/kg every 2 weeks with ipilimumab 1 mg/kg every 6 weeks. All arms demonstrated manageable safety and durable responses. with ORR up to 32%. Arm A showed the most favorable survival, with a median OS of 22.8 months compared to 12.5 and 12.7 months in Arms B and C, respectively (98). Based on these findings, the Arm A regimen received accelerated FDA approval in 2020 as second-line therapy for HCC (**Table 2**). An extended 5-year follow-up study confirmed its sustained efficacy with a 60-month OS rate of 29% (102).

The combination of nivolumab and ipilimumab was further evaluated as first-line treatment for unresectable HCC in the phase 3 CheckMate 9DW trial. Results demonstrated that the combination significantly improved OS compared to lenvatinib or sorafenib (median OS: 23.7 vs 20.6 months; HR 0.79, 95% CI 0.65–0.96; p=0.018). At 24 and 36 months, OS rates were 49% and 38% with the combination, versus 39% and 24% with lenvatinib or sorafenib, respectively. The ORR was 36%, and median PFS was 9.1 months. Grade 3–4 treatment-related adverse events occurred in 41% of patients receiving the combination (99). These results led to FDA approval of the combination as a first-line treatment for unresectable HCC in 2025 (**Table 2**).

#### Durvalumab + tremelimumab in HCC

Dual checkpoint inhibition with durvalumab (PD-L1 inhibitor) and tremelimumab (CTLA-4 inhibitor) has been evaluated in unresectable hepatocellular carcinoma (HCC) through a phase 1/2 study followed by a confirmatory phase 3 trial (**Table 6**). The phase 1/2 study assessed two dosing regimens: a single priming dose of tremelimumab 300 mg plus durvalumab 1,500 mg every 4 weeks (T300+D), and tremelimumab 75 mg every 4 weeks combined with durvalumab 1,500 mg every 4 weeks (T75+D). The T300+D regimen demonstrated superior efficacy, with an ORR of 24.0% versus 9.5%, median OS of 18.73 versus 11.30 months, and 18-month OS rates of 52.0% versus 34.7%. Safety was manageable, with grade ≥3 adverse events in 37.8% of patients (100). Based on these findings, the T300+D regimen (also referred to as STRIDE) was selected for the open-label phase 3 HIMALAYA trial, which compared STRIDE to sorafenib in systemic therapy-naive patients with unresectable HCC. STRIDE achieved an ORR of 20.1%, median OS of 16.4 months, and 18-, 24-, and 36-month OS rates of 48.7%, 40.5%, and 30.7%, respectively. Although median PFS was modest at 3.8 months, the regimen significantly improved OS versus sorafenib (HR 0.78, 96.02% CI 0.65–0.93; p=0.0035), supporting its clinical benefit. Grade 3–4 adverse events occurred in 50.5% of patients, consistent with the known toxicity profile (101). These results led to FDA approval of the STRIDE regimen for first-line treatment of advanced HCC in 2022 (**Table 2**). Separately, a phase 2 study investigated STRIDE in patients with unresectable HCC and chronic active hepatitis B. The study demonstrated an ORR of 25% and supported the regimen’s feasibility when administered concurrently with anti-HBV medication (103).

**Table 6:** Clinical studies in hepatocellular carcinoma.

*Patient group*	*Treatment*	*Participants (n)*	*Trial, ID*	*Outcomes*	*Reference*
*Unresectable HCC*	Nivolumab + ipilimumab	148 (Arm A^1^=50, B^2^ =49, C^3^=49)	Phase 1/2, NCT01658878 (CheckMate 040)	**Investigator-assessed ORR:** 32% | 27% | 29% **ORR (BICR):** 32% | 31% | 31% **Median OS:** 22.8 months | 12.5 months | 12.7 months **12-month OS rate:** 61% | 55% | 50% **24-month OS rate:** 48% | 30% | 42%	Yau et al., 2020 (98)
*Unresectable HCC*	Nivolumab + ipilimumab	335	Phase 3, NCT04039607 (CheckMate 9DW)	**ORR:** 36% **Median OS:** 23.7 months **24-month OS rate:** 49% **36-month OS rate:** 38% **Median PFS:** 9.1 months **18-month PFS rate:** 34% **24-month PFS rate:** 28% **Grade 3–4 AE:** 41%	Yau et al., 2025 (99)
*Unresectable HCC*	Durvalumab + tremelimumab	159 (T300+D^4^=75, T75+D^5^=84)	Phase 1/2, NCT02519348	**ORR:** 24.0% | 9.5% **Median PFS:** 2.17 months | 1.87 months **Median OS:** 18.73 months | 11.30 months **18-month OS rate:** 52.0% | 34.7% **Grade ≥3 AE:** 37.8% | 24.4%	Kelley et al., 2021 (100)
*Unresectable HCC*	Durvalumab + tremelimumab	393	Phase 3, NCT03298451 (HIMALAYA)	**Investigator-assessed ORR:** 20.1% **Median OS:** 16.4 months **18-month OS rate:** 48.7% **24-month OS rate:** 40.5% **36-month OS rate:** 30.7% **Median PFS:** 3.8 months **Any-grade AEs:** 97.4% **Grade 3–4 AEs:** 50.5%	Abou-Alfa et al., 2022 (101)

^1^Nivolumab 1 mg/kg plus ipilimumab 3 mg/kg every 3 weeks (4 doses) followed by nivolumab 240 mg intravenously every 2 weeks.

^2^Nivolumab 3 mg/kg plus ipilimumab 1 mg/kg every 3 weeks (4 doses) followed by nivolumab 240 mg intravenously every 2 weeks.

^3^Nivolumab 3 mg/kg every 2 weeks plus ipilimumab 1 mg/kg every 6 weeks.

^4^Tremelimumab 300 mg plus durvalumab 1,500 mg every 4 weeks.

^5^Tremelimumab 75 mg plus durvalumab 1,500 mg every 4 weeks. AE, adverse event; BICR, blinded independent central review; ORR, objective response rate; OS, overall survival; PFS, progression-free survival.

### Lung cancer

#### Nivolumab + ipilimumab in NSCLC

Dual ICI with nivolumab and ipilimumab has been extensively studied in non-small cell lung carcinoma (NSCLC) (**Table 7**) (104–108, 114–122). Initial evaluation as first-line therapy for advanced NSCLC occurred in a phase 1 trial comparing two dosing regimens: nivolumab every 2 weeks with ipilimumab every 12 or 6 weeks. ORRs were 47% and 38%, with grade 3–4 adverse events in 37% and 33%, respectively. Notably, tumors with PD-L1 expression ≥1% showed higher response rates (57% in both cohorts) (104). A phase 2 study confirmed the safety and efficacy of low-dose ipilimumab (1 mg/kg every 6 weeks) plus nivolumab (3 mg/kg every 2 weeks), and found that response rates were higher in patients with tissue tumor mutational burden (tTMB) ≥10 mutations per megabase (mut/Mb), regardless of PD-L1 status (114). Subsequently, a phase 3 trial in previously untreated stage IV or recurrent NSCLC demonstrated that among patients with high tTMB (≥10 mut/Mb), the combination achieved an ORR of 45.3%, 1-year PFS of 42.6%, and median PFS of 7.2 months (HR 0.58 vs chemotherapy; p<0.001). In contrast, patients with low tTMB had shorter median PFS with the combination compared to chemotherapy (3.2 vs 5.5 months) (105). OS was similar across PD-L1 subgroups. In PD-L1 ≥1% tumors, median OS was 17.1 months, with 1- and 2-year OS rates of 62.6% and 40.0%; in PD-L1 <1% tumors, median OS was 17.2 months, with OS rates of 60% and 40.4%, respectively. However, the combination provided an OS benefit over chemotherapy regardless of PD-L1 expression (HR 0.79 [97.72% CI, 0.65–0.96] and 0.62 [95% CI, 0.48–0.78]) (106). These findings led to the FDA approval of nivolumab (3 mg/kg) plus ipilimumab (1 mg/kg) for first-line treatment of advanced NSCLC with PD-L1 ≥1% in 2020 (**Table 2**). Sustained survival benefits were reported in 4- and 5-year follow-ups, with OS rates of 29% and 24% for PD-L1 ≥1%, and 24% and 19% for PD-L1 <1%, respectively (115, 116). Patient-reported outcomes also favored the combination, with delayed symptom deterioration and improved health-related quality of life compared to chemotherapy (117).

**Table 7:** Clinical studies in lung cancer.

*Patient group*	*Treatment*	*Participants (n)*	*Trial, ID*	*Outcomes*	*Reference*
*Untreated advanced NSCLC*	Nivolumab + ipilimumab	77 (NIVO+IPI12^1^=38, NIVO+IPI6^2^=39)	Phase 1, NCT01454102 (CheckMate 012)	**ORR:** 47% | 38% **Median PFS:** 8.1 months | 3.9 months **24-week PFS rate:** 68% | 47% **Median OS:** Not calculated | Not calculated **1 year OS rate:** Not calculated | 69% **Grade 3–4 AEs:** 37% | 33%	Hellmann et al., 2017 (104)
*Untreated metastatic or recurrent NSCLC (TMB)*	Nivolumab + ipilimumab	139	Phase 3, NCT02477826 (CheckMate 227)	**ORR:** 45.3% **1-year PFS rate irrespective of TMB:** 30.9% **1-year PFS rate in high TMB:** 42.6% **Median PFS irrespective of TMB:** 4.9 months **Median PFS in high TMB:** 7.2 months **Grade 3–4 AEs:** 31.2%	Hellmann et al., 2018 (105)
*Untreated metastatic or recurrent NSCLC (PD-L1 expression)*	Nivolumab + ipilimumab	583 (PD-L1 ≥1%=396, PD-L1 <1 = 187)	Phase 3, NCT02477826 (CheckMate 227)	**ORR:** 35.9% | - **Median OS:** 17.1 months | 17.2 months **1-year OS rate:** 62.6% | 60% **2-year OS rate:** 40.0% | 40.4% **Grade 3–4 AE:** 32.8%	Hellmann et al., 2019 (106)
*Platinum pretreated NSCLC*	Nivolumab + ipilimumab	125	Phase 3, NCT02785952 (Lung-MAP S1400I)	**ORR:** 18% **Median OS:** 10 months **1-year OS:** 45% **2-year OS:** 28% **Median PFS:** 3.8 months **1-year PFS:** 17% **2-year PFS:** 12% **Grade 3–4 AE:** 39.5%	Gettinger et al., 2021 (107)
*Resectable NSCLC*	Nivolumab + ipilimumab	113	Phase 3, NCT02998528 (CheckMate 816)	**Median EFS:** 54.8 months **3-year EFS:** 56% **pCR:** 20.4% **MPR:** 28.3% **Median OS:** Not reached **3-year OS:** 73% **Grade 3–4 AE:** 14%	Awad et al., 2025 (108)
*Untreated metastatic NSCLC* *(PD-L1 ≥25%)*	Durvalumab + tremelimumab	163	Phase 3, NCT024532828 (MYSTIC)	**Median OS:** 11.9 months **Median OS (bTMB ≥20 mutations per megabase):** 21.9 months **24-month OS:** 35.4% **Median PFS:** 3.9 months **12-month PFS:** 25.8% **Grade ≥ 3 AE:** 22.9%	Rizvi et al., 2020 (109)
*Untreated metastatic NSCLC (bTMB ≥20 mut/Mb)*	Durvalumab + tremelimumab	410	Phase 3, NCT02542293 (NEPTUNE)	**ORR:** 27.5% **Median OS:** 11.7 months **24-month OS:** 26.1% **Median PFS:** 4.2 months **12-month PFS:** 25.6% **Garde 3–4 AE:** 20.7%	De Castro et al., 2022 (110)
*Pretreated NSCLC (PD-L1 TC ≥25%)*	Durvalumab + tremelimumab	174	Phase 3, NCT02352948 (ARCTIC)	**ORR:** 14.9% **Median OS:** 11.5 months **12-month OS:** 49.5% **Median PFS:** 3.5 months **6-month PFS:** 31.5% **12-month PFS:** 20.6% **Grade 3–4 TRAE:** 22.0%	Planchard et al., 2020 (111)
*Untreated metastatic NSCLC*	Pembrolizumab + ipilimumab	284	Phase 3, NCT03302234 (KEYNOTE-598)	**ORR:** 45.4% **Median OS:** 21.4 months **12-month OS**: 63.6% **Median PFS:** 8.2 months **Grade ≥ 3 AE:** 62.4%	Boyer et al., 2021 (112)
*Pretreated recurrent SCLC*	Nivolumab + ipilimumab	96	Phase 1/2, NCT01928394 (CheckMate 032)	**ORR:** 21.9% **Median PFS:** 1.5 months **12 months PFS rate:** 11.9% **Median OS:** 4.7 months **24 months OS rate:** 16.9% **Grade 3–4 AEs:** 37.5%	Ready et al., 2020 (113)

^1^Nivolumab every 2 weeks plus ipilimumab every 12 weeks.

^2^Nivolumab every 2 weeks plus ipilimumab every 6 weeks. AE, adverse event; bTMB, blood tumor mutational burden; MPR, major pathological response; ORR, objective response rate; OS, overall survival; pCR, pathological complete response; PFS, progression-free survival; TMB, tumor mutational burden; TRAE, treatment-related adverse event.

Separately, a phase 3 trial evaluating nivolumab plus ipilimumab in patients with advanced NSCLC previously treated with platinum-based chemotherapy showed no significant improvement in outcomes. The combination yielded an ORR of 18%, median OS of 10 months, and median PFS of 3.8 months. HR for death (0.87; p = 0.34) and progression (0.80; p = 0.09) were not statistically significant (107).

In the neoadjuvant setting for resectable NSCLC, the combination has shown greater efficacy than nivolumab alone. A phase 2 trial reported higher rates of major pathological response (MPR: 50% vs 24%) and pathological complete response (pCR: 38% vs 10%), along with enhanced immune infiltration and memory (118). Additionally, a phase 3 study comparing the combination to chemotherapy demonstrated improved event-free survival (54.8 vs 20.9 months), higher pCR (20.4% vs 4.6%) and MPR (28.3% vs 14.8%) rates, and better tolerability (Grade 3–4 AE: 14% vs 36%). HR for disease progression, recurrence, or death was 0.77 (108).

#### Durvalumab + tremelimumab in NSCLC

The combination of durvalumab and tremelimumab has also been explored in NSCLC across multiple studies (**Table 7**) (109–111, 123–126). A phase 1b trial found that durvalumab 20 mg/kg every 4 weeks plus tremelimumab 1 mg/kg was tolerable and showed antitumor activity regardless of PD-L1 expression (123). In a phase 3 trial of first-line treatment for metastatic NSCLC with PD-L1 ≥25%, the combination yielded a median OS of 11.9 months and PFS of 3.9 months, with no significant improvement over chemotherapy (HR for death 0.85, p=0.20). However, exploratory analysis identified a blood TMB (bTMB) ≥20 mut/Mb as a potential predictive marker, with improved median OS of 21.9 months (HR 0.49, 95% CI 0.32–0.74) (109). Another phase 3 trial similarly found a trend toward better outcomes at higher bTMB levels, though results were not statistically significant. In patients with bTMB ≥20, the HR for death was 0.71 (p=0.081), with median OS of 11.7 vs 9.1 months and 2-year OS rates of 26.1% vs 13.6%, compared to standard chemotherapy. Lower or intermediate bTMB cutoffs (≥12 or ≥16) showed smaller effects (HRs 0.94 and 0.87), suggesting a trend toward improved outcomes with increasing bTMB levels. Additional investigation is required to validate bTMB as a predictive biomarker and define the subset of patients most likely to benefit from this combination (110).

The combination has also been tested as second-line therapy in advanced NSCLC. A phase 1b study evaluated its safety and efficacy in patients previously treated with PD-1/PD-L1 inhibitors. While the safety profile was manageable (grade 3–4 AEs in 28.2%), efficacy was limited, with response rates of 5.3% in PD-1/PD-L1-refractory and 0% in PD-1/PD-L1-relapsed patients (124). Similarly, a phase 2 trial reported minimal activity, with ORRs of 7% in the primary resistance cohort and 0% in the acquired resistance cohort to prior anti–PD-1/PD-L1 therapy. Median PFS was approximately 2 months and median OS around 7.7 months in both groups (125).

A phase 3 study evaluated the combination as third-line or later treatment in metastatic NSCLC with PD-L1 TC expression <25%. Although the results did not reach statistical significance, numerical improvements in OS and PFS were observed compared to standard of care (SoC). HR for death and progression were 0.80 (p=0.109) and 0.77 (p=0.056), respectively. Median OS was 11.5 months with the combination vs 8.7 months with SoC, while median PFS was 3.5 months in both groups. Interestingly, in a *post hoc* exploratory analysis of the PD-L1 TC <1% subgroup (n=72), HR for OS and PFS were 0.59 and 0.55, respectively (111).

#### Pembrolizumab + ipilimumab in NSCLC

A randomized, double-blind phase 3 trial compared pembrolizumab plus ipilimumab to pembrolizumab alone in treatment-naïve metastatic NSCLC patients with PD-L1 Tumor Proportion Score ≥50% and no EGFR or ALK alterations. Median OS was similar (21.4 vs 21.9 months), as was PFS (8.2 vs 8.4 months), while grade ≥3 adverse events were more frequent with the combination (62.4% vs 50.2%). The study concluded that adding ipilimumab did not improve outcomes and increased toxicity compared to pembrolizumab monotherapy (112).

A phase 1/2 study evaluated the combination as second-line or later therapy in advanced NSCLC, reporting an ORR of 30%, median PFS of 4.1 months, and OS of 10.9 months. Grade 3–4 adverse events occurred in 29% of patients. Authors concluded that the combination demonstrated signs of antitumor activity in heavily pretreated advanced NSCLC but was associated with notable toxicity (127).

#### Nivolumab + ipilimumab in SCLC

Concurrent nivolumab and ipilimumab have been evaluated in recurrent SCLC after prior chemotherapy in a phase 1/2 study (113, 128). Nivolumab 1 mg/kg plus ipilimumab 3 mg/kg showed the highest activity in the initial nonrandomized cohort and was advanced to the randomized phase (128). In this cohort, the combination achieved an ORR of 21.9% and median OS of 4.7 months, while nivolumab monotherapy showed an ORR of 11.6% and median OS of 5.7 months. Two-year OS rates were similar (16.9% vs. 17.9%), but grade 3–4 TRAEs were more frequent with the combination (37.5% vs. 12.9%). Although response rates were higher with the combination, OS was comparable and toxicity was greater (113).

In a phase 3 trial, nivolumab plus ipilimumab was evaluated as maintenance therapy in extensive-disease SCLC following first-line chemotherapy. The combination did not significantly improve OS versus placebo (median OS: 9.2 vs. 9.6 months; HR 0.92; p = 0.37), although PFS improved (HR 0.72). Grade 3–4 TRAEs occurred in 52.2% of patients (129). Similarly, a phase 2 trial in limited-disease SCLC, consolidation nivolumab plus ipilimumab following chemoradiotherapy and protocol amendment-1 failed to improve PFS (median 10.7 vs. 14.5 months; HR 1.02; p = 0.93) or OS (HR 0.95; p = 0.82) compared to observation. High-grade toxicity was common, with grade ≥3 AEs in 62% of patients (130).

### Mesothelioma

#### Nivolumab + ipilimumab in mesothelioma

The combination of nivolumab and ipilimumab in patients with relapsed malignant pleural mesothelioma was first evaluated in two phase 2 trials (**Table 8**). The first trial, a single-center, single-arm study, enrolled patients who had progressed after at least one line of platinum-based chemotherapy. The results showed an ORR of 38%, median PFS of 6.2 months, and a 12-month OS rate of 64%, with grade 3–4 adverse events reported in 38% of patients (131). In a separate multicenter, randomized, non-comparative phase 2 trial, patients who had failed one or two prior lines of chemotherapy were assigned to receive either nivolumab monotherapy or the combination. Among those receiving the combination, the ORR was 27.8%, median OS was 15.9 months, and 12-month OS rate was 58.1%, with 26.2% experiencing grade 3–4 toxicity (132). Based on encouraging results from these studies, a randomized, multicenter phase 3 trial was launched to assess the combination in the first-line setting. In this study, patients with unresectable malignant pleural mesothelioma were randomized to receive either nivolumab plus ipilimumab or standard chemotherapy. Compared to platinum-based chemotherapy, nivolumab plus ipilimumab significantly improved OS (median OS: 18.1 vs. 14.1 months; HR 0.74 [96.6% CI 0.60–0.91]; p=0.0020), with a 2-year OS rate of 41% versus 27% and manageable toxicity (30% grade 3–4 adverse events) (133). These findings led to FDA approval of the regimen in 2020 as a first-line treatment for unresectable malignant pleural mesothelioma (**Table 2**). A subsequent 3-year follow-up analysis showed 3-year OS rates of 23% versus 15% and PFS rates of 14% versus 1%, further supporting the long-term benefit of the combination (121).

**Table 8:** Clinical studies in mesothelioma.

*Patient group*	*Treatment*	*Participants (n)*	*Trial, ID*	*Outcomes*	*Reference*
*Recurrent malignant pleural mesothelioma*	Nivolumab + ipilimumab	35	Phase 2, NCT03048474 (INITIATE)	**ORR:** 38% **Median PFS:** 6.2 months **6-month PFS rate:** 50% **Median OS:** not reached **12-month OS rate:** 64% **Grade 3–4 AEs:** 38%	Disselhorst et al., 2019 (131)
*Relapsed malignant pleural mesothelioma*	Nivolumab + ipilimumab	62	Phase 2, NCT02716272	**ORR:** 27.8% **Median PFS:** 5.6 months **12 months PFS rate:** 22.6% **Median OS:** 15.9 months **12 months OS rate:** 58.1% **Grade 3–4 AEs:** 26.2%	Scherpereel et al., 2019 (132)
*Unresectable malignant pleural mesothelioma*	Nivolumab + ipilimumab	303	Phase 3, NCT02899299 (CheckMate 743)	**ORR:** 40% **Median OS:** 18.1 months **1-year OS rate:** 68% **2-year OS rate:** 41% **Median PFS: 6.8** months **2-year PFS rate:** 16% **Grade 3–4 AEs**: 30%	Baas et al., 2021 (133)
*Malignant mesothelioma*	Durvalumab + tremelimumab	40	Phase 2, NCT02588131	**irORR: 28%** **ORR: 25%** **Median PFS:** 8.0 months **Median OS:** 16.6 months **1-year OS rate:** 62% **Grade 3–4 AE:** 18%	Calabro et al., 2018 (134)
*Malignant mesothelioma*	Durvalumab + tremelimumab	11	Phase 2, NCT02592551	**Median OS:** Not reached **Grade ≥3 AE:** 27%	Lee et al., 2023 (135)

AE, adverse event; irORR, immune-related objective response rate; ORR, objective response rate; OS, overall survival; PFS, progression-free survival.

#### Durvalumab + tremelimumab in mesothelioma

Dual immune checkpoint blockade with durvalumab and tremelimumab has been explored in two phase 2 studies in malignant mesothelioma (**Table 8**). The first, an open-label, non-randomized trial, investigated the combination in patients with unresectable pleural or peritoneal mesothelioma. The regimen showed encouraging efficacy and manageable toxicity, with an ORR of 25%, median PFS of 8.0 months, median OS of 16.6 months, and grade 3–4 adverse events in 18% of patients. Although not designed to directly compare treatment lines, a *post-hoc* analysis revealed objective response rates of 33% in first-line and 25% in second-line settings (134). The second study assessed the immune effects of neoadjuvant durvalumab plus tremelimumab versus durvalumab alone in resectable malignant pleural mesothelioma. Both regimens led to CD8+ T cell infiltration within tumors without changing CD8/Treg ratios. However, combination therapy was associated with improved survival, with median OS not reached at a 34.1-month follow-up, compared to 14 months in the monotherapy group (135). Together, these studies suggest that the durvalumab–tremelimumab combination is active and well tolerated across neoadjuvant, first-line, and second-line settings in malignant mesothelioma, warranting further investigation in larger, randomized trials.

### Esophageal squamous cell carcinoma

#### Nivolumab + ipilimumab in ESCC

A phase 3 trial evaluated nivolumab plus ipilimumab as first-line treatment for advanced ESCC (**Table 9**). The combination showed superior efficacy over chemotherapy alone, particularly in patients with PD-L1 positive tumors (PD-L1 expression ≥1%). In the overall population, ORR was similar (28% vs 27%), but the combination led to more complete responses (11% vs 6%) and longer median OS (12.7 vs 10.7 months). Among patients with PD-L1 positive tumors, the combination achieved higher ORR (35% vs 20%), more complete responses (18% vs 5%), and longer OS (13.7 vs 9.1 months), with comparable PFS (4.0 months vs 4.4 months). Safety was consistent with known profiles, with 32% experiencing grade 3–4 adverse events (136). Based on these results, the FDA approved the regimen for advanced esophageal squamous-cell carcinoma in 2022 (**Table 2**).

**Table 9:** Clinical studies in esophageal squamous cell carcinoma.

*Patient group*	*Treatment*	*Participants (n)*	*Trial, ID*	*Outcomes*	*Reference*
*Advanced ESCC*	Nivolumab + ipilimumab	325	Phase 3, NCT03143153 (CheckMate 648)	**ORR in PD-L1 positive:** 35% **ORR overall:** 28% **Median OS in PD-L1 positive:** 13.7 months **Median OS overall:** 12.7 months **12-month OS rate in PD-L1 positive:** 57% **Median PFS in PD-L1 positive:** 4.0 months **Median PFS overall:** Not tested **Grade 3–4 AEs:** 32%	Doki et al., 2022 (136)
*Advanced ESCC*	Durvalumab + tremelimumab	59	Phase 1, NCT01938612	**ORR overall:** 20.3% **ORR in PD‐L1 TC ≥ 25%:** 50% **ORR in PD‐L1 TC < 25%:** 12.2% **ORR in PD-L1 TC ≥ 1%:** 24.2% **ORR in PD-L1 TC < 1%:** 6.3% **Median PFS:** 2.9 months **Median OS:** 7.9 months **12-month OS rate:** 35.3% **Grade ≥ 3 AE:** 13.6%	Doki et al., 2022 (137)

AE, adverse event; ORR, objective response rate; OS, overall survival; PFS, progression-free survival; TC, tumor cells.

#### Durvalumab + tremelimumab in ESCC

The combination of durvalumab and tremelimumab in the treatment of advanced ESCC was tested in a phase 1 study (**Table 9**). While not designed for a direct comparison, the combination showed greater efficacy than durvalumab alone. Patients receiving the combination had higher ORR (20.3% vs 7.1%), longer median PFS (2.9 vs 1.4 months), longer median OS (7.9 vs 5.2 months), and a higher 12-month OS rate (35.3% vs 22.3%). Consistent with prior findings, higher PD-L1 expression was associated with improved response rates, with ORRs of 50% for tumors expressing ≥25%, 12.2% for <25%, 24.2% for ≥1%, and 6.3% for <1%. Authors concluded that the combination showed promising activity and a tolerable safety profile, warranting further investigation in future studies (137).

#### Breast cancer

The combination of nivolumab and ipilimumab was evaluated in two phase 2 trials in patients with breast cancer (**Table 10**) (138, 139). One study focused on metaplastic breast cancer and was conducted as a prospective, open-label, multicenter trial assessing safety and efficacy. The combination achieved an ORR of 18%, with a median PFS of 2 months and median OS of 12 months. Notably, responses were durable—lasting over 28, 33, and 34 months—and occurred in tumors with low TMB, low tumor-infiltrating lymphocytes (TILs), and no PD-L1 expression (138). The other study investigated the immune-activating effects of the combination in early-stage triple-negative breast cancer. In contrast, a correlation was observed between clinical response and high TIL levels, prompting an exploratory cohort of patients with ≥50% TILs. In this group, 53% (8/15) achieved a major pathological response, including 33% with pathological complete response. The authors concluded that neoadjuvant administration without chemotherapy demonstrates promising activity and warrants further investigation (139).

**Table 10:** Clinical studies in breast cancer.

*Patient group*	*Treatment*	*Participants (n)*	*Trial, ID*	*Outcomes*	*Reference*
*Unresectable or metastatic metaplastic breast cancer*	Nivolumab + ipilimumab	17	Phase 2, NCT02834013 (DART)	**ORR:** 18% **Median PFS**: 2 months **Median OS:** 12 months **Grade 3–4 AEs:** 18%	Adams et al., 2021 (138)
*Early-stage triple-negative breast cancer*	Nivolumab + ipilimumab	15	Phase 2, NCT03815890 (BELLINI)	**Immune activation:** 60% **MPR:** 53% **pCR:** 33%	Nederlof et al., 2024 (139)

AE, adverse event; MPR, major pathological response; ORR, objective response rate; OS = overall survival; pCR, pathological complete response; PFs, progression-free survival.

### Glioblastoma

Combination of nivolumab and ipilimumab for the treatment of recurrent glioblastoma was first tested in a phase 1 study (**Table 11**). The study investigated multiple dose regimens of the combination therapy, concurrently comparing with monotherapy with nivolumab. Combination therapies (NIVO1+IPI3 and NIVO3+IPI1) showed limited efficacy (ORR 0% and 10%, median PFS 2.1 and 1.5 months, median OS 7.3 and 9.2 months) and high occurrence of grade 3–4 adverse events (90% vs 30%). Nivolumab monotherapy demonstrated better efficacy (ORR 11%, median PFS 1.9 months, median OS 10.4 months) and a more favorable safety profile (0% grade 3–4 adverse events) (140). Another phase 1 study investigated administration of nivolumab and ipilimumab intracerebrally as intravenous administration yielded low activity. The study reported a median PFS of 2.7 months, median OS of 8.7 months, 6-months OS rate of 74.1%, 12-months OS rate of 40.7% and 24-months OS rate of 27% (141).

**Table 11:** Clinical studies in glioblastoma.

*Patient group*	*Treatment*	*Participants (n)*	*Trial, ID*	*Outcomes*	*Reference*
*Recurrent glioblastoma*	Nivolumab + ipilimumab	40 (NIVO1+IPI3^1^=10, NIVO3+IPI1^2^=20)	Phase 1, NCT02017717	**ORR:** 0% | 10% **Median PFS:** 1.5 months | 2.1 months **Median OS:** 9.2 months | 7.3 months **Grade 3–4 AEs:** 90% | 30%	Omuro et al., 2018 (140)
*Recurrent glioblastoma*	Intracerebral nivolumab + ipilimumab	27	Phase 1, NCT03233152	**Median PFS:** 2.7 months **Median OS**: 8.7 months **6 months OS rate:** 74.1% **12 months OS rate:** 40.7% **24 months OS rate:** 27%	Duerinck et al., 2021 (141)
*Newly diagnosed glioblastoma*	Nivolumab + ipilimumab	15	Phase 1, NCT03425292	**Median PFS:** 1.3 months **Median OS:** 19.3 months **Grade 3–4 AE:** 9%	Kesari et al., 2024 (142)

^1^Nivolumab 1 mg/kg plus ipilimumab 3 mg/kg.

^2^Nivolumab 3 mg/kg plus ipilimumab 1 mg/kg. AE, adverse event; ORR, objective response rate; OS, overall survival; PFS, progression-free survival.

A phase 1 trial also evaluated the combination in newly diagnosed glioblastoma in the neoadjuvant setting prior to standard radiotherapy. The study reported a median PFS of 1.3 months and median OS of 19.3 months. Notably, median OS was 35.7 months in patients with MGMT promoter methylation, compared to 12.6 months in unmethylated cases. Grade 3–4 adverse events occurred in 9% of patients (142).

### Head and neck squamous cell carcinoma

#### Nivolumab + ipilimumab in HNSCC

Dual immunotherapy with nivolumab and ipilimumab was assessed as a first-line treatment for patients with recurrent or metastatic HNSCC in a phase 3 trial, comparing its efficacy to the EXTREME regimen (cetuximab plus platinum-based chemotherapy and fluorouracil, followed by cetuximab maintenance). The study did not meet its primary OS endpoints in either the overall population or the PD-L1 Combined Positive Score (CPS) ≥20 subgroup. Median OS was similar between arms (13.9 vs 13.5 months overall; 17.6 vs 14.6 months in CPS ≥20), without statistical significance. ORR was higher with EXTREME in the overall population (36.8% vs 24.2%) and comparable in the CPS ≥20 subgroup (36.1% vs 34.1%). The authors concluded that dual immunotherapy did not significantly improve outcomes over standard treatment (143). Additionally, a phase 2 study assessed nivolumab plus ipilimumab vs nivolumab alone in platinum-refractory or platinum-sensitive recurrent/metastatic HNSCC. The study did not meet its primary end point of ORR benefit. In platinum-refractory patients, ORR was 13.2% vs 18.3% with monotherapy; in platinum-sensitive patients, 20.3% vs 29.5%, respectively (147).

A phase 1b/2 study investigated neoadjuvant nivolumab plus ipilimumab in patients with advanced or recurrent HNSCC eligible for curative-intent surgery. Major pathological responses were observed in 35% of patients, and none of these patients experienced recurrence during a median follow-up of 24 months after surgery (148). Similarly, a phase 2 study in treatment-naive oral cavity squamous cell carcinoma reported encouraging activity, with volumetric responses in 53%, any pathological response in 73%, and major or complete pathological responses in 20% (149).

#### Durvalumab + tremelimumab in HNSCC

Durvalumab plus tremelimumab has been investigated in recurrent or metastatic HNSCC (**Table 12**). A phase 2 study in patients with PD-L1–low or negative tumors evaluated durvalumab with or without tremelimumab. Both arms demonstrated clinical activity (ORR: 9.2% vs. 7.8%; median OS: 6.0 vs. 7.6 months for combination vs. monotherapy), with manageable toxicity (grade 3–4 AEs: 15.8% vs. 12.3%) (144). In a phase 3 trial of patients progressing after platinum-based therapy, durvalumab with or without tremelimumab was compared to standard-of-care (SoC: cetuximab, taxane, methotrexate, or fluoropyrimidine). No significant differences were observed in both OS (HR 1.04, p = 0.76; median 6.5 months) or PFS (HR 1.09, p = 0.54; median 2.0 months) between the combination and SoC (145). Another phase 3 trial compared the combination to the EXTREME regimen in treatment-naive patients. Again, the combination failed to improve OS, both in patients with high PD-L1 expression (HR 1.05) and in the overall population (HR 1.04) (146). Notably, although neither study was designed for direct comparison, durvalumab monotherapy appeared to show more favorable activity and tolerability than the addition of tremelimumab (145, 146).

**Table 12:** Clinical studies in head and neck squamous cell carcinoma.

*Patient group*	*Treatment*	*Participants (n)*	*Trial, ID*	*Outcomes*	*Reference*
*Recurrent or metastatic HNSCC*	Nivolumab + ipilimumab	472	Phase 3, NCT02741570 (CheckMate 651)	**ORR:** 24.2% **ORR in PD-L1 CPS ≥ 20:** 34.1% **Median OS:** 13.9 months **Median OS in PD-L1 CPS ≥ 20:** 17.6 months **Median PFS:** 3.3 months **Median PFS in PD-L1 CPS ≥ 20:** 5.4 months **Grade 3–4 AEs:** 28.2%	Haddad et al., 2022 (143)
*Recurrent or metastatic HNSCC (PD-L1-low/negative (TC<25%))*	Durvalumab + tremelimumab	133	Phase 2, NCT02319044 (CONDOR)	**ORR:** 7.8% **ORR in PD-L1 TC<1%:** 7.4% **ORR in PD-L1 TC<10%:** 6.8% **Median PFS:** 2.0 months **6-month PFS rate:** 13.7% **Median OS:** 7.6 months **12-month OS rate:** 37% **Grade 3–4 AE:** 15.8%	Siu et al., 2019 (144)
*Previously platinum treated recurrent or metastatic HNSCC*	Durvalumab + tremelimumab	247	Phase 3, NCT02369874 (EAGLE)	**ORR:** 18.2% **Median OS:** 6.5 months **12-month OS:** 30.4% **18-month OS:** 21.0% **24-month OS:** 13.3% **Median PFS:** 2.0 months **Grade ≥3 TRAE:** 16.3%	Ferris et al., 2020 (145)
*Recurrent or metastatic HNSCC*	Durvalumab + tremelimumab	413	Phase 3, NCT02551159 (KESTREL)	**ORR overall:** 21.8% **ORR in PD-L1 TC ≥50%:** 25.3% **Median OS overall:** 10.7 months **Median OS in PD-L1 TC ≥50%:** 11.2 months **12-month OS rate:** 46.5% **18-month OS rate:** 30.7% **24-month OS rate:** 22.9% **Median PFS overall:** 2.8 months **Median PFS in PD-L1 TC ≥50%:** 2.8 months **Grade 3–4 AEs:** 19.1%	Psyrri et al., 2022 (146)

AE, adverse event; CPS, combined positive score; ORR, objective response rate; OS, overall survival; PFS, progression-free survival; TC, tumor cells; TRAE, treatment-related adverse event.

The feasibility of neoadjuvant durvalumab plus tremelimumab in locally advanced, resectable HNSCC was evaluated in a randomized phase 2 study. The combination was well tolerated and did not delay surgery. Notably, distant recurrence-free survival was significantly improved compared to durvalumab monotherapy, with 4-year OS rates of 83.1% versus 67.5%, respectively (150).

### Merkel cell carcinoma

The combination of nivolumab and ipilimumab was evaluated in two studies for advanced Merkel cell carcinoma (**Table 13**). In one open-label, randomized phase 2 trial, patients received the combination either as first-line therapy or after prior immune checkpoint inhibitor (ICI) treatment. Promising efficacy was observed in both groups, with ICI-naïve patients showing more favorable outcomes: ORR was 100% vs 42%, median PFS was not reached vs 4.2 months, and median OS was not reached vs 14.9 months, in ICI-naïve and previously treated patients, respectively (151). The second study, a nonrandomized, multicohort phase I/II trial, compared the combination to nivolumab monotherapy. While both treatment arms demonstrated durable responses, the combination failed to show superior efficacy and, notably, underperformed compared to monotherapy. ORR was similar (58% vs 60%), but median PFS (8.4 vs 21.3 months) and median OS (29.8 vs 80.7 months) were substantially shorter in the combination arm. Based on these findings, the authors recommended conducting a randomized trial comparing nivolumab plus ipilimumab directly with nivolumab monotherapy to definitively assess the findings (152).

**Table 13:** Clinical studies in Merkel cell carcinoma.

*Patient group*	*Treatment*	*Participants (n)*	*Trial, ID*	*Outcomes*	*Reference*
*Unresectable or advanced Merkel cell carcinoma*	Nivolumab + ipilimumab	25 (ICI-naïve = 13, Prior ICI = 12)	Phase 2, NCT03071406	**ORR:** 100% | 42% **Median PFS:** Not reached | 4.2 months **Median OS:** Not reached | 14.9 months **Grade 3–4 AEs overall:** 36%	Kim et al., 2022 (151)
*Recurrent or metastatic Merkel cell carcinoma*	Nivolumab + ipilimumab	43	Phase 1/2, NCT02488759 (CheckMate 358)	**ORR:** 58% **Median PFS:** 8.4 months **Median OS:** 29.8 months **Grade 3–4 AEs:** 47%	Bhatia et al., 2025 (152)

AE, adverse event; ICI, immune checkpoint inhibitor; ORR, objective response rate; OS, overall survival; PFS, progression-free survival.

### Ovarian cancer

#### Nivolumab + ipilimumab in ovarian cancer

The efficacy of nivolumab plus ipilimumab versus nivolumab monotherapy in recurrent or persistent epithelial ovarian cancer was assessed in an open-label, randomized phase 2 trial (**Table 14**). The combination demonstrated superior outcomes, with an ORR of 31.4% vs 12.2%, median PFS of 3.9 vs 2.0 months, 6-month PFS rate of 25.5% vs 16.3%, and median OS of 28.1 vs 21.8 months, respectively. HR for progression and death were significantly lower with the combination (0.53 and 0.79, respectively). Toxicities were consistent with expectations and manageable, with 66.7% of patients experiencing grade 3–4 adverse events, prompting the authors to recommend further investigation (153).

**Table 14:** Clinical studies in ovarian cancer.

*Patient group*	*Treatment*	*Participants (n)*	*Trial, ID*	*Outcomes*	*Reference*
*Recurrent or persistent ovarian cancer*	Nivolumab + ipilimumab	51	Phase 2, NCT02498600	**ORR:** 31.4% **Median PFS:** 3.9 months **6 months PFS rate:** 25.5% **Median OS:** 28.1 months **Grade 3–4 AEs:** 66.7%	Zamarin et al., 2020 (153)
*Recurrent gynecologic malignancies*	Intraperitoneal nivolumab + ipilimumab	23	Phase 1b, NCT03508570	**Median PFS:** 2.7 months **1-year PFS rate:** 13% **Median OS:** Not reached **1-year OS rate:** 77% **2-year OS rate:** 68% **Grade 3–4 AE:** 8.7%	Knisely et al., 2024 (154)
*Recurrent platinum-resistant ovarian cancer*	Durvalumab + tremelimumab	23	Phase 2, NCT03026062	**Stable disease:** 4.4% **Partial response:** 8.7% **Median PFS:** 1.9 months **Median OS:** 7.3 months **Grade >3 AE:** 30.4%	Hinchcliff et al., 2024 (155)

AE, adverse event; ORR, objective response rate; OS, overall survival; PFS, progression-free survival.

A phase 1b study evaluated intraperitoneal administration of nivolumab and ipilimumab in recurrent gynecologic cancers with peritoneal carcinomatosis. Initial cohorts received nivolumab monotherapy, and subsequent cohorts received the combination. The combined results showed promising 1- and 2-year OS rates of 77% and 68%, respectively. Median OS was not reached. Notably, the incidence of grade 3–4 adverse events was significantly low at 8.7%, supporting the feasibility of intraperitoneal delivery (154).

#### Durvalumab + tremelimumab in ovarian cancer

An open-label, randomized phase 2 trial evaluated durvalumab plus tremelimumab compared to tremelimumab alone (**Table 14**). The study included a heavily pretreated population with platinum-resistant high-grade serous ovarian cancer. While the combination arm showed a higher partial response rate (8.7% vs 0%), the clinical benefit rate, which included stable disease, was greater in the monotherapy arm (31.6% vs 4.4%). Median PFS was similar (1.87 vs 1.84 months), and OS was slightly longer with monotherapy (10.6 vs 7.3 months). The authors concluded that there was no significant difference in efficacy and no added benefit with combination therapy (155).

### Prostate cancer

A phase 2 trial evaluated the combination of nivolumab (PD-1 inhibitor) plus ipilimumab (CTLA-4 inhibitor) in metastatic prostate cancer patients with AR-V7 expression (**Table 15**). Among those with measurable soft-tissue disease, the ORR was 25%. Median PFS and OS were 3.7 and 8.2 months, respectively. Notably, outcomes were better in tumors with DNA repair deficiency (DRD), showing an ORR of 40% vs 0% in DRD-negative tumors, with hazard ratios for progression and death of 0.31 and 0.41 (156). Since AR-V7+ patients comprise a small subset of metastatic castration-resistant prostate cancer (mCRPC), a phase 2 study assessed the combination in unselected mCRPC, both pre- and post-chemotherapy. The study reported ORRs of 25% and 10%, with median OS of 19.0 and 15.2 months in chemotherapy-naïve and chemotherapy-experienced patients, respectively (157). Both authors concluded that their findings warrant further investigation of nivolumab and ipilimumab in prostate cancer through large prospective trials.

**Table 15:** Clinical studies in prostate cancer.

*Patient group*	*Treatment*	*Participants (n)*	*Trial, ID*	*Outcomes*	*Reference*
*AR-V7-expressing metastatic prostate cancer*	Nivolumab + ipilimumab	15	Phase 2, NCT02601014	**ORR:** 25% **Median PFS:** 3.7 months **Median OS:** 8.2 months **Grade 3–4 AEs:** 46%	Boudadi et al., 2018 (156)
*Metastatic castration-resistant prostate cancer (mCRPC)*	Nivolumab + ipilimumab	90 (pre-chemo=45, post-chemo=45)	Phase 2, NCT02985957 (CheckMate 650)	**ORR:** 25% | 10% **Median OS:** 19.0 months | 15.2 months **Grade 3–4 AEs:** 42.2% | 53.3%	Sharma et al., 2020 (157)

AE, adverse event; ORR, objective response rate; OS, overall survival; PFS, progression-free survival.

### Sarcoma

#### Nivolumab + ipilimumab in sarcoma

Nivolumab, alone or in combination with ipilimumab, was evaluated in patients with locally advanced, unresectable, or metastatic sarcoma who had received at least one prior line of systemic therapy. This was done in an open-label, multi-center, randomized phase 2 study. The analysis showed an ORR of 5% with nivolumab alone and 16% with the combination. Median PFS was 1.7 months vs 4.1 months, median OS was 10.7 months vs 14.3 months, and 12-month OS rates were 40.4% vs 54.6%, respectively. The authors concluded that nivolumab monotherapy had limited activity, while the combination demonstrated more encouraging efficacy and warrants further evaluation in larger randomized studies (158).

A prospective, open-label phase 2 trial evaluated the combination in metastatic or unresectable angiosarcoma. The ORR was 25%, 6-month PFS rate 38%, and median OS was not reached. Grade 3–4 adverse events occurred in 25% of patients. Despite the small sample size, notably higher responses were seen in cutaneous tumors (60%), warranting further study (159).

The efficacy and safety of the combination was evaluated in a prospective, single-arm phase 2 study of patients with classical Kaposi sarcoma who had received at least one prior line of systemic therapy. The results demonstrated promising efficacy, with radiologic, clinical, and metabolic ORRs of 87%, 78%, and 93%, respectively. Median PFS was not reached; 6- and 12-month PFS rates were 76.5% and 58.8%. Grade 3–4 adverse events occurred in 22% of patients and were considered manageable. Interestingly, most patients had low or negative PD-L1 expression, TMB, and TILs, regardless of treatment response. The authors concluded that the combination represents a promising treatment option for progressive classical Kaposi sarcoma (160).

A phase 1/2 trial in pediatric sarcomas established nivolumab 3 mg/kg plus ipilimumab 1 mg/kg as the recommended phase 2 dose in children based on safety and pharmacokinetics. Although overall responses were limited, two patients with Ewing and rhabdomyosarcoma showed durable partial responses, prompting the authors to recommend further study in these subtypes (162).

#### Durvalumab + tremelimumab in sarcoma

The combination of durvalumab and tremelimumab was evaluated in a single-center, phase 2 trial in patients with recurrent or metastatic sarcoma previously treated with systemic therapy (**Table 16**). The study reported an ORR of 12%, median PFS of 2.8 months, and median OS of 21.6 months. The 12-week, 12-month, and 24-month PFS rates were 49%, 28%, and 15%, while OS rates at 12 and 24 months were 65% and 49%, respectively. Grade 3–4 adverse events occurred in 37% of patients. Notably, the authors observed encouraging activity in alveolar soft-part sarcoma, with a 12-week PFS rate of 80% and the highest response rates by irRC (50%) and irRECIST (40%). Although based on a small sample, the authors concluded that these findings support further investigation in this histologic subtype (161).

**Table 16:** Clinical studies in sarcoma.

*Patient group*	*Treatment*	*Participants (n)*	*Trial, ID*	*Outcomes*	*Reference*
*Locally advanced, unresectable or metastatic sarcoma*	Nivolumab + ipilimumab	41	Phase 2, NCT02500797	**ORR**: 16% **Median PFS**: 4.1 months **Median OS**: 14.3 months **12 months OS rate**: 54.6% **Grade 3–4 AEs**: 14%	D’angelo et al., 2018 (158)
*Metastatic or unresectable angiosarcoma*	Nivolumab + ipilimumab	16	Phase 2, NCT02834013	**ORR:** 25% **6-month PFS rate:** 38% **Median OS:** Not reached **Grade 3–4 AEs:** 25%	Wagner et al., 2021 (159)
*Previously treated classical Kaposi sarcoma*	Nivolumab + ipilimumab	18	Phase 2, NCT03219671	**Radiologic ORR:** 87% **Clinical ORR:** 78% **Metabolic ORR:** 93% **Median PFS:** Not reached **6-month PFS rate:** 76.5% **1-year PFS rate:** 58.8% **Grade 3–4 AEs:** 22%	Zer et al., 2022 (160)
*Advanced or metastatic soft tissue and bone sarcoma*	Durvalumab + tremelimumab	57	Phase 2, NCT02815995	**ORR:** 12% **Median PFS:** 2.8 months **12-week PFS rate:** 49% **12-month PFS rate:** 28% **24-month PFS rate:** 15% **Median OS:** 21.6 months **12-month OS rate:** 65% **24-month OS rate:** 49% **Grade 3–4 AEs:** 37%	Somaiah et al., 2022 (161)

AE, adverse event; ORR, objective response rate; OS, overall survival; PFS, progression-free survival.

### Urothelial carcinoma

#### Nivolumab + ipilimumab in UC

Dual checkpoint blockade with nivolumab and ipilimumab in metastatic urothelial carcinoma was first evaluated in a phase 1/2 study, later followed by a phase 2 study (**Table 17**). The initial study assessed safety and efficacy across three dosing regimens in patients with previously treated unresectable or metastatic disease: nivolumab 3 mg/kg monotherapy (NIVO3), nivolumab 3 mg/kg plus ipilimumab 1 mg/kg (NIVO3+IPI1), and nivolumab 1 mg/kg plus ipilimumab 3 mg/kg (NIVO1+IPI3). Among these, NIVO1+IPI3 showed the most promising activity, achieving the highest ORR (38%), longest median OS (15.3 months), and longest PFS (4.9 months), compared to NIVO3 (25.6%, 9.9 months, 2.8 months) and NIVO3+IPI1 (26.9%, 7.4 months, 2.6 months). Grade 3–4 adverse events were slightly more frequent with NIVO1+IPI3 (39.1%) than with NIVO3 (26.9%) or NIVO3+IPI1 (30.8%) (163). In the following phase 2 study, NIVO1+IPI3 was evaluated as second-line treatment in patients with platinum-pretreated metastatic urothelial carcinoma. Patients were given induction monotherapy with nivolumab, and those with stable or progressive-disease at week 8 received the combination regimen. The overall ORR was 33%, with a median OS of 7.6 months and PFS of 1.9 months. Notably, ORR was substantially higher in PD-L1 positive tumors (46%) compared to PD-L1 negative tumors (24%), suggesting greater benefit in this subgroup. The safety profile was consistent, with grade 3–4 adverse events in 36% of patients. The authors proposed that NIVO1+IPI3 may serve as a feasible “rescue” strategy for early non-responders or those with late progression, particularly in PD-L1 positive disease (164).

**Table 17:** Clinical studies in urothelial carcinoma.

*Patient group*	*Treatment*	*Participants (n)*	*Trial, ID*	*Outcomes*	*Reference*
*Platinum pre-treated locally advanced or metastatic UC*	Nivolumab + ipilimumab	196 (NIVO3+IPI1^1^=104, NIVO1+IPI3^2^=92)	Phase 1/2, NCT01928394 (CheckMate 032)	**ORR:** 26.9% | 38% **Median PFS:** 2.6 months | 4.9 months **12 months PFS rate:** 22.6% | 25.9% **Median OS:** 7.4 months | 15.3 months **12 months OS rate:** 38.3% | 56.9% **Grade 3–4 AEs:** 30.8% | 39.1%	Sharma et al., 2019 (163)
*Platinum pre-treated metastatic UC*	Nivolumab + ipilimumab	83	Phase 2, NCT03219775 (TITAN-TCC)	**ORR: 3**3% **ORR in PD-L1 positive:** 46% **ORR in PD-L1 negative:** 24% **Median OS:** 7.6 months **6-month OS rate:** 56% **12-month OS rate**: 39% **18-month OS rate:** 36% **Median PFS:** 1.9 months **6-month PFS rate:** 28% **12-month PFS rate:** 21% **18-month PFS rate**: 18% **Grade 3–4 AEs:** 36%	Grimm et al., 2023 (164)
*Locoregionally advanced UC*	Nivolumab + ipilimumab	30 (NIVO3+IPI1^1^ = 15, NIVO1+IPI3^2^=15)	Phase 1b, NCT03387761 (NABUCCO)	**pCR:** 7% | 43% **No residual disease:** 29% | 57% **Grade ≥3 AE:** 20% | 33%	Van Dorp et al., 2023 (165)
*Locoregionally advanced UC*	Nivolumab + ipilimumab	24	Phase 1b, NCT03387761 (NABUCCO)	**pCR:** 46% **No residual disease:** 58%	Van Dijk et al., 2020 (166)
*Platinum-resistant metastatic UC*	Durvalumab + tremelimumab	168	Phase 1, NCT02261220	**ORR:** 20.8% **ORR in PD-L1 ≥25%:** 29.4% **ORR in PD-L1 <25%:** 15.1% **Median PFS:** 1.9 months **Median PFS in PD-L1 ≥25%:** 3.5 months **Median PFS in PD-L1 <25%:** 1.8 months **6-month PFS rate:** 25.4% **Median OS**: 9.5 months **Median OS in PD-L1 ≥25%:** 18.9 months **Median OS in PD-L1 <25%:** 8.0 months **Grade 3–4 AE:** 28.6%	Balar et al., 2018 (167)
*Previously untreated unresectable, locally advanced or metastatic UC*	Durvalumab + tremelimumab	342	Phase 3, NCT02516241 (DANUBE)	**ORR:** 36% **ORR in high PD-L1:** 47% **Median OS:** 15.1 months **Median OS in high PD-L1:** 17.9 months **Median PFS:** 3.7 months **Median PFS in high PD-L1:** 4.1 months **Grade 3–4 AE:** 27%	Powles et al., 2020 (168)
*Cisplatin-ineligible operable high-risk UC*	Durvalumab + tremelimumab	28	Phase 1, NCT02812420	**1-year OS:** 88.8% **pCR:** 37.5% **Downstaging (p ≤ T1N0):** 58.3% **Grade 3–4 AE:** 21%	Gao et al., 2020 (169)

^1^Nivolumab 3 mg/kg plus ipilimumab 1 mg/kg.

^2^Nivolumab 1 mg/kg plus ipilimumab 3 mg/kg. AE, adverse event; ORR, objective response rate; OS, overall survival; pCR, pathological complete response; PFS, progression-free survival

A phase 1b study investigated the feasibility of nivolumab plus ipilimumab in the neoadjuvant setting for locoregionally advanced urothelial carcinoma. The initial cohort received NIVO1+IPI3, achieving a pathologic complete response (pCR) in 46% and absence of residual muscle-invasive disease in 58% of patients (166). A subsequent cohort compared NIVO1+IPI3 with NIVO3+IPI1. Outcomes with NIVO1+IPI3 were consistent (pCR 43%, no residual disease 57%) and notably superior to NIVO3+IPI1 (pCR 7%, no residual disease 29%) (165). Interestingly, the authors noted that pCR appeared independent of baseline CD8+ T-cell presence or T-effector signatures, though absence of pre-surgical circulating tumor DNA in plasma was potentially predictive of PFS. Both studies concluded that neoadjuvant dual immunotherapy shows promise and warrants further investigation in randomized trials (165, 166).

#### Durvalumab + tremelimumab in UC

A multicenter phase 1 study evaluated the combination of durvalumab and tremelimumab patients with platinum-resistant metastatic urothelial carcinoma (**Table 17**). The results demonstrated an ORR of 20.8%, median PFS of 1.9 months, and median OS of 9.5 months. Grade 3–4 adverse events occurred in 28.6% of patients, indicating a manageable safety profile. Notably, patients with PD-L1 expression ≥25% experienced higher ORR (29.4% vs 15.1%) and longer median OS (18.9 vs 8.0 months) compared to those with lower expression. However, clinical activity was still observed across PD-L1 subgroups, supporting the authors’ conclusion that the regimen offered a manageable safety profile and encouraging antitumor activity regardless of PD-L1 status (167).

The combination was also assessed as a first-line treatment for unresectable, locally advanced, or metastatic urothelial carcinoma in a randomized, multicenter phase 3 trial. It showed an ORR of 36%, median OS of 15.1 months, and median PFS of 3.7 months, with slightly improved outcomes in patients with high PD-L1 expression (ORR 47%, OS 17.9 months, PFS 4.1 months). Compared to standard platinum-based chemotherapy, the combination yielded an OS hazard ratio of 0.85 (95% CI 0.72–1.02; p = 0.075), and 0.74 (95% CI 0.59–0.93) in the high PD-L1 subgroup—both not meeting prespecified thresholds for statistical significance. As such, the study concluded that the combination did not provide a survival advantage over standard chemotherapy, regardless of PD-L1 status. However, the combination did demonstrate greater activity (in terms of OS and ORR) than durvalumab monotherapy (168).

Safety and efficacy of neoadjuvant durvalumab plus tremelimumab were evaluated in a phase 1 study in cisplatin-ineligible patients with operable high-risk urothelial carcinoma. The combination led to a pathological complete response in 37.5% and tumor downstaging to pT1 or less in 58% of patients who completed surgery. Grade 3–4 adverse events occurred in 21% of patients (169).

## Summary

The complementary mechanisms of action of PD-1/PD-L1 and CTLA-4 inhibitors have demonstrated synergistic effects, improving response rates, overall survival, and progression-free survival in various cancer types. The FDA has approved the combination of nivolumab (PD-1 inhibitor) and ipilimumab (CTLA-4 inhibitor) for the treatment of BRAF V600 wild-type unresectable or metastatic melanoma, metastatic RCC, colorectal cancer with MSI-H/dMMR, unresectable HCC, advanced NSCLC, unresectable malignant pleural mesothelioma, and advanced or metastatic ESCC. In addition, the combination of durvalumab (PD-L1 inhibitor) and tremelimumab (CTLA-4 inhibitor) has been approved for unresectable HCC (**Table 1**). The majority of clinical studies have evaluated nivolumab plus ipilimumab, demonstrating promising efficacy in other malignancies such as breast cancer, ovarian cancer, prostate cancer, sarcoma, and urothelial carcinoma. However, limited clinical benefit has been observed in glioblastoma, HNSCC, and Merkel cell carcinoma. Similarly, durvalumab plus tremelimumab has shown efficacy in NSCLC, mesothelioma, and ESCC, but with limited benefit in HNSCC and ovarian cancer. Other combinations, such as pembrolizumab (PD-1 inhibitor) with ipilimumab, have also been evaluated in a limited number of trials. Further research is needed to investigate other approved anti-PD-1/PD-L1 and CTLA-4 combinations—including cemiplimab, dostarlimab, retifanlimab, toripalimab, tislelizumab, avelumab, and cosibelimab.

Combination ICI therapy targeting PD-1/PD-L1 and CTLA-4 generally exhibits greater efficacy than monotherapy but is also associated with increased frequency and severity of treatment-related adverse events. These toxicities can affect virtually any organ system, with the most commonly involved being the endocrine system (e.g., thyroid dysfunction, hypophysitis, adrenal insufficiency), skin (e.g., rash, pruritus), gastrointestinal tract (e.g., colitis, diarrhea), liver (e.g., hepatitis), and lungs (e.g., pneumonitis) (170, 171). Although rare, neurologic and cardiac toxicity carry the highest fatality rates (172, 173). Overall, the spectrum of adverse events appears relatively consistent across tumor types (174, 175). However, some reports suggest a slightly increased frequency of organ-specific toxicities depending on the tumor type, such as pneumonitis in NSCLC and vitiligo in melanoma. The underlying mechanisms for these associations remain unclear (174, 176–178). Toxicity profiles also differ between single-agent PD-1/PD-L1 and CTLA-4 blockade. CTLA-4 inhibitors are more commonly linked to colitis, hypophysitis, and rash, whereas PD-1/PD-L1 inhibitors are more often associated with thyroid dysfunction and pneumonitis. Thus, both the incidence and type of immune related AEs are influenced by the specific agent and its dose (170, 174, 176, 177, 179). To optimize safety while preserving efficacy, various dosing regimens have been investigated. Notably, higher doses of CTLA-4 inhibitors have been associated with increased toxicity and modestly enhanced efficacy (62, 63, 81, 98, 100, 128, 140, 180). Interestingly, the optimal dose of nivolumab and ipilimumab appears to vary by cancer type. For instance, in melanoma, RCC, NSCLC, and several other cancers, the NIVO3+IPI1 regimen (3 mg/kg nivolumab + 1 mg/kg ipilimumab) has demonstrated favorable efficacy with manageable toxicity (62, 81, 104). Conversely, in HCC and SCLC, the NIVO1+IPI3 regimen has shown superior outcomes (98, 128). The variations in effective doses and responses underscore the intricate distinctions in the TME among different cancer subtypes. Moreover, in glioblastoma, HNSCC, and Merkel cell carcinoma, monotherapy has shown superior outcomes compared to combination therapy, adding another layer of complexity to therapeutic decision-making (140, 147, 152).

Despite progress, the mechanisms underlying why some patients exhibit complete and durable responses while others experience relapse remain poorly understood. Therefore, identifying reliable predictive biomarkers is a major research focus. Currently, PD-L1 expression, TMB, and MSI/dMMR status are FDA-approved biomarkers, though each has shown limited or inconsistent predictive value. While PD-L1 expression correlates with response in cancers such as NSCLC, ESCC, and urothelial carcinoma, it has shown inconsistent or no correlation in others, including breast cancer, glioblastoma, and HNSCC (105, 106, 114, 136, 139, 146, 164). Moreover, meaningful responses have also been observed in PD-L1-negative patients (111). TMB has emerged as another potential biomarker, with higher mutational burden generally associated with improved ICI efficacy, as supported by a retrospective analysis of 27 tumor types (181). In NSCLC, the CheckMate 227 trial demonstrated that patients with TMB ≥10 mut/Mb had superior responses to nivolumab plus ipilimumab, regardless of PD-L1 status (105). Nevertheless, some patients with low TMB also derive benefit, underscoring its limitations as a standalone biomarker (182, 183). MSI and dMMR have also shown value as predictive biomarkers, particularly in colorectal, gastric, and endometrial cancers (184). However, MSI-H tumors often harbor numerous passenger mutations without functional relevance, indicating that microsatellite count alone is insufficient (185). Furthermore, only a small subset of patients with cancer have MSI-H tumors (186). TILs are also widely recognized as a potential biomarker and are generally associated with favorable prognosis; however, only a subset of CD8+ TILs can recognize tumor-specific neoantigens, contributing to heterogeneous clinical responses (182, 187). Emerging biomarkers related to the gut microbiome and the TME, including hypoxia, inflammatory cytokines and transcription factors, and the collagen architecture of the extracellular matrix, are also being explored based on theoretical and mechanistic rationale, though their clinical utility remains to be validated (188–190). Continued research is needed to refine patient selection strategies and optimize therapeutic outcomes with ICI-based immunotherapy.

## Conclusion

The combination of PD-1/PD-L1 and CTLA-4 inhibitors has led to significant improvements in clinical outcomes across multiple cancer types, offering enhanced response rates and survival benefits. However, these benefits come with a higher incidence of adverse events, making toxicity management a critical concern. Optimizing dosing regimens has shown promise in maintaining efficacy while reducing toxicity, though the ideal balance varies by cancer type. The heterogeneity of treatment responses highlights the complexity of the tumor microenvironment and reinforces the need for a deeper mechanistic understanding. Identifying robust and reliable biomarkers remains a key priority to improve patient selection and maximize therapeutic benefit.
